# A machine learning model to predict the risk of 30-day readmissions in patients with heart failure: a retrospective analysis of electronic medical records data

**DOI:** 10.1186/s12911-018-0620-z

**Published:** 2018-06-22

**Authors:** Sara Bersche Golas, Takuma Shibahara, Stephen Agboola, Hiroko Otaki, Jumpei Sato, Tatsuya Nakae, Toru Hisamitsu, Go Kojima, Jennifer Felsted, Sujay Kakarmath, Joseph Kvedar, Kamal Jethwani

**Affiliations:** 1 Partners Connected Health Innovation, Partners HealthCare, 25 New Chardon St., Suite 300, Boston, MA 02114 USA; 2 Massachusetts General Hospital, Boston, MA USA; 3 Harvard Medical School, Boston, MA USA; 4 Research and Development Group, Hitachi, Ltd, Tokyo, Japan

**Keywords:** Heart failure, Machine learning, Deep learning, Deep unified networks, Readmission reduction, Value-based care

## Abstract

**Background:**

Heart failure is one of the leading causes of hospitalization in the United States. Advances in big data solutions allow for storage, management, and mining of large volumes of structured and semi-structured data, such as complex healthcare data. Applying these advances to complex healthcare data has led to the development of risk prediction models to help identify patients who would benefit most from disease management programs in an effort to reduce readmissions and healthcare cost, but the results of these efforts have been varied. The primary aim of this study was to develop a 30-day readmission risk prediction model for heart failure patients discharged from a hospital admission.

**Methods:**

We used longitudinal electronic medical record data of heart failure patients admitted within a large healthcare system. Feature vectors included structured demographic, utilization, and clinical data, as well as selected extracts of un-structured data from clinician-authored notes. The risk prediction model was developed using deep unified networks (DUNs), a new mesh-like network structure of deep learning designed to avoid over-fitting. The model was validated with 10-fold cross-validation and results compared to models based on logistic regression, gradient boosting, and maxout networks. Overall model performance was assessed using concordance statistic. We also selected a discrimination threshold based on maximum projected cost saving to the Partners Healthcare system.

**Results:**

Data from 11,510 patients with 27,334 admissions and 6369 30-day readmissions were used to train the model. After data processing, the final model included 3512 variables. The DUNs model had the best performance after 10-fold cross-validation. AUCs for prediction models were 0.664 ± 0.015, 0.650 ± 0.011, 0.695 ± 0.016 and 0.705 ± 0.015 for logistic regression, gradient boosting, maxout networks, and DUNs respectively. The DUNs model had an accuracy of 76.4% at the classification threshold that corresponded with maximum cost saving to the hospital.

**Conclusions:**

Deep learning techniques performed better than other traditional techniques in developing this EMR-based prediction model for 30-day readmissions in heart failure patients. Such models can be used to identify heart failure patients with impending hospitalization, enabling care teams to target interventions at their most high-risk patients and improving overall clinical outcomes.

**Electronic supplementary material:**

The online version of this article (10.1186/s12911-018-0620-z) contains supplementary material, which is available to authorized users.

## Background

As of 2013, heart failure affects (HF) 5.7 million Americans with annual costs of $30.7 billion [1]. It is one of the leading causes of hospitalization in the United States (US), particularly in patients aged 65 years and above [2]. 1 in 4 heart failure patients are readmitted within 30 days of discharge, and risk-adjusted all-cause readmission rates declined only slightly from 2009 (20%) to 2012 (19%) in Medicare beneficiaries [3].

In recognition of this troubling trend, the Center for Medicare & Medicaid Services (CMS), the largest payer for medical services in the US, instituted penalties for hospitals with excess readmissions for heart failure. This policy change resulted in a shift from the traditional model of fee-for-service to value-based care [4]. In response, major hospitals and healthcare systems have been implementing strategies to decrease risk of readmission following hospital discharge. The outcomes thus far are encouraging: a pre-post analysis of changes in readmission rates before and after Medicare’s Hospital Readmissions Reduction Program (HRRP) showed that HF-specific risk-standardized readmissions decreased after HRRP by 84.7 per 10,000 discharges per year (compared with an increase of 5.1 per 10,000 discharges per year before HRRP) [5]. However, as of 2013, the rate of all-cause readmission for heart failure index admissions was still 23.5%, the highest rate of readmission following an index stay for a high-volume condition (higher than that for chronic obstructive pulmonary disease (20.0%), pneumonia (15.5%), and acute myocardial infarction (14.7%) [6].

To control rising hospitalization cost and improve outcomes in HF management, an area of promise is predictive analytics. Within healthcare, prediction models may be used to develop disease- or event-related risk assessment tools. While advances in big data solutions now allow for storage, management, and mining of large volumes of structured and semi-structured data such as healthcare data, leveraging these advances to build prediction models in healthcare poses challenges. Healthcare data is notoriously noisy and heterogeneous [7], existing in multiple databases across even a single healthcare delivery system. However, big data analytics tend to be tolerant of poor data quality, though applications are naturally more valid and clinically useful when applied to higher quality data [8]. Additionally, methods such as data mining and machine learning can make use of all variables available in a data set without presumed associations between variables or predictive power of any particular variables. These methods allow for identification of potentially highly predictive variables that otherwise may have gone unexplored using more traditional methods such as logistic regression.

Despite challenges, and in light of the great need to improve quality of care and patient outcomes, effort continues in developing prediction models to assess patient risk for complications or adverse events [9]. Overall, the literature reports modest performances of predictive models for readmissions in heart failure patients, with few models demonstrating an area under the curve (AUC) of ≥0.70 [9, 10]. Citing the mixed results of readmission prediction models for HF patients which use traditional methods, recent studies [7, 11–14] are testing whether machine learning methods, which can take into account higher-order and nonlinear interactions between predictors, might demonstrate higher performance. Results show modest improvements over traditional statistical methods when compared directly, though AUCs demonstrate a comparable range of performance (0.54 [12] to 0.78 [13]). The mixed results of big data models highlight the need for further research to add clarity to the existing body of literature, as well as more approaches to demonstrate the ability to implement these models (if appropriate) into clinical practice, and provide more evidence that the application of these models can translate into improved healthcare quality, outcomes, and lower healthcare costs.

A great majority of efforts to develop predictive models frequently rely on structured data. Given the volume and richness of data available in unstructured clinical notes or reports, machine learning models may benefit from leveraging text mining tools to enhance the model. In this paper, we describe the development of a risk prediction model using structured and unstructured electronic medical record (EMR) data to predict the risk of 30-day readmission in HF patients, capitalizing on admission data available at discharge.

## Methods

### Aims

The primary aim of this study was to develop a machine learning model to predict the risk of all-cause 30-day readmission for HF patients.

Our secondary aim was to estimate the potential healthcare utilization cost savings that could be achieved if a telemonitoring intervention is selectively offered to patients at high risk of 30-day readmission.

### Design

This retrospective study used de-identified structured and unstructured patient data from the EMR of a large healthcare delivery network, applying a deep learning method to develop a model to predict the risk of 30-day readmission for HF patients discharged from a hospital admission. Records included in the derivation dataset were from HF patients who were discharged alive from an inpatient hospital admission between 2014 and 2015.

### Settings

All study data were generated from inpatient and outpatient encounters which took place within the Partners Healthcare System (PHS), a not-for-profit network of seven major hospitals, including two large academic centers in the Boston Metro area, and several community health centers across eastern Massachusetts. To be included, all patients had to have had at least one HF hospital encounter and have been discharged alive from an inpatient encounter (i.e. admission) at least once from any PHS facility.

### Data sources

There are 2 sources of data for this study: i) Enterprise Data Warehouse (EDW): The EDW aggregates structured data from multiple PHS source systems – clinical, operational, financial, and claims – to create a consistent view of data collected across these systems; ii) Research Patient Data Repository (RPDR): The RPDR is a centralized clinical data warehouse that gathers structured and unstructured data from multiple hospital EMR systems and stores it in one place. Data content includes demographic and clinical data only; it does not include operational, financial, or claims data.

### Data selection

To be considered a ‘HF patient’ in this study, we considered patients who were aged 18 years or older and diagnosed with heart failure as designated by any hospital encounter with a principal diagnosis of HF (International Classification of Diseases (ICD)-9 Codes: 402.01, 402.11, 402.91, 404.01, 404.03, 404.11, 404.13, 404.91, 428.xx) anytime between fiscal years 2011-2015 (Oct 2010 – Sept 2015). Medical records were extracted for those patients who were discharged alive from an inpatient admission at least once anytime during the study period: fiscal years 2014-2015 (Oct 2013 – Sept 2015).

Qualifying patients were identified using the above criteria with no methods applied to balance the readmission ratio of the patient populations. We then extracted the following structured data elements: demographics, hospital utilization information, diagnoses, procedures, labs, and medications; and the following unstructured data elements: physician notes and discharge summaries.

### Outcomes

The outcome of interest was all-cause 30-day readmission. Every hospitalization was designated an index admission with potential for a readmission. An index admission may be flagged as having a 30-day readmission if the number of days between discharge of the index admission and its subsequent readmission is ≤30 days. Not all index admissions have readmissions. Readmissions may themselves be index admissions if the readmission is followed by a subsequent readmission within ≤30 days of discharge. Within-hospital transfer, patient leaving against medical advice, and planned admissions (e.g. chemotherapy, radiation, dialysis, birth/delivery) were all excluded from the readmission counts.

### Statistical plan

Data from the EDW and RPDR were extracted and managed using structured query language (SQL) within Microsoft SQL Server Management Studio 2016 (SSMS). All data was de-identified using the “Safe harbor” method: removal of 18 identifiers recommended by the Health Insurance Portability and Accountability Act (HIPAA), including names, dates, contact information, ages > 89, ID numbers, etc. of patients or relatives, employers, or household members of patients, as well as device identifiers, serial numbers, and policy numbers. Text-mining techniques were used to de-identify unstructured data using a combination of SQL Server Management Studio and SweetScape Text Editor 010. Statistical analysis of baseline patient social and clinical demographics was completed using R version 3.2.2 in RStudio. Vector data generation was completed using Apache Spark 2.1.0. Text processing was completed using Python v2.7.13 and Stanford core NLP library v.3.5.2. Python v.2.7.13 was used to build three prediction models: logistic regression (Scikit-learn v0.18.1), gradient boosting (XGBoost v0.6), deep learning (Keras v1.2.1 and Theano v0.9.0 backend). Hyperopt v0.1 library was used to optimize hyper parameters of the prediction methods [15].

### Feature vector generation and model building

Feature vectors for the prediction model were calculated by medication information code mapping, HF-relevant well-known factors (WKF) mapping, text data selection, and variable coding. To build a high accuracy readmission prediction model, we developed a new architecture of deep learning: deep unified networks (DUNs). The details of each process are explained in the subsections below.

#### Medication information code mapping

All names of prescribed drugs were mapped to anatomical therapeutic chemical (ATC) classification codes to have the active ingredient as an independent variable of the feature vector. ATC classification codes have five levels based on efficacy, action site, and scientific features. The variables of the feature vector were composed of the fifth level of the ATC classification code that expresses the active ingredient. First, RxNorm codes (a naming system developed by the National Library of Medicine which normalizes US generic and brand drug names) were mapped by matching drug names from the medication master data set to RxNorm drug names. The RxNorm codes were then converted to fifth level ATC classification codes by using information about the relationship between RxNorm codes and ATC classification codes. Using this strategy, we allocated ATC classification codes to 88.9% of the drug names present in the medication master data set.

#### Heart failure relevant well-known factors mapping

Our prediction model used WKFs relevant to HF according to the American College of Cardiology Foundation (ACCF) and American Heart Association (AHA) guidelines, and a previous study by Sun J et al. [16, 17]. Codes to create the WKFs were extracted from three types of data elements: diagnoses, clinical laboratory tests (labs), and medications.

For diagnoses, diseases associated with HF (e.g. pneumonia, hypertension, diabetes mellitus) were identified from the ACCF/AHA guideline and mapped by their ICD-9 diagnosis codes. For clinical laboratory tests, brain natriuretic peptide (BNP) and N-terminal pro b-type natriuretic peptide (NT-proBNP) codes were used as well-known biomarkers of HF, in addition to codes from tests frequently used during hospital stays: hematological codes such as hemoglobin, white blood cell count, and alanine aminotransferase (ALT), and urinalysis codes. All test names were mapped to logical observation identifiers names and codes (LOINC). For medications, drugs associated with HF, such as aldosterone antagonists and thiazides, were identified according to the ACCF/AHA guideline and Sun J et al., followed by mapping based on the ATC classification.

#### Text data selection and pre-processing

The data set contained two types of unstructured patient notes – physician notes and discharge summaries – which contain information about the cause of admission, the patients’ hospital course, and discharge conclusions and instructions. Text data were divided into sections by content header. The contents of each document vary with each section. We selected sections which were present in 50% or more of notes posted during inpatient admissions. Physicians’ notes contained sections pertaining to patients’ social history (e.g. smoking/drinking history, description of family network). Discharge summaries contained sections pertaining to hospital course and treatment; allergic reactions, intolerances and sensitivities; history of present illness and reason for hospitalization, and significant findings.

Text data were converted numerically before the application of machine learning. The bag-of-words (BOW) model was employed to express the text as feature vectors of numerals. The feature vectors regard the frequency of appearance of each word in each document and the presence/absence of its appearance (binary). Words were transformed to standard word form using lemmatization techniques. A part-of-speech (POS) filtering technique extracted specific words including nouns, adjectives, and prepositions. Because words with low frequencies of appearance in the entire document could have less contribution to the prediction model, we deleted words that appeared less than or equal to 5 times.

#### Feature variable coding

Each variable was stored in a unique element of a feature vector. Target periods, data sources, and value expressions for each data type were set as shown in Table 1.

**Table 1:** Target periods and value expressions of each data type

Data Type	Data Source	Target periods	Value expression
Demographics	EDW	*Patient-level, no applicable target period*	Binary (0 or 1); Continuous/discrete value
Admissions	EDW	Admission date to discharge date	Continuous/discrete value
Diagnoses	EDW	2 years pre-discharge date to discharge date	Binary of occurrence in the target period
Labs	EDW	Admission date to 1 week after admission date; 1 week before discharge date to discharge date	Number of occurrences in the target period ^a^
Medications	EDW/RPDR	Admission date to discharge date	Number of occurrences in the target period
Procedures	EDW	Admission date to discharge date	Number of occurrences in the target period
Notes	RPDR	Admission date to discharge date ^b^	Binary of occurrence in the target period ^c^

^a^ abnormal occurrences, ^b^ except Social History = penultimate to admission date, ^c^ except Allergies = number of occurrences in the target period

Demographic data included patient characteristic and socioeconomic variables such as age, race, marital status, level of education, employment status, and median income. Admissions data included variables such as the dates of admission and discharge, length of stay, readmission status, principal diagnosis, admission source, and discharge disposition. Diagnosis data included the date of the encounter (inpatient admission or outpatient visit), number of diagnoses, and ICD-9 codes for up to 20 diagnosis positions where applicable. Labs data included the name of the lab test, LOINC, date of the order, text or numeric result of the test, and out-of-range status of the result. Medications data consisted of prescription (not claims) information detailing name of the medication, date of the prescription update, and dose and strength of the drug. Procedures data included the date of the encounter (inpatient admission or outpatient visit), number of procedures, and ICD-9 codes for up to 20 procedure positions where applicable. Notes data included the type of note (discharge summary or physician note), date the note was initiated, subject of the note, and the text extract corresponding to the section of interest (e.g. reason for hospitalization, hospital course, social history, allergies, etc.).

1-of K expression converted the quantitative variables to elements of the feature vector [18]. To deal with missing data, interpolation techniques such as zero, median, and mean filtering were tested and found to be comparable, thus missing values in each variable were treated as zero. Additionally, to improve the numerical stability of the prediction modeling, each variable was rescaled in the range from 0.0 to 1.0 by using min-max normalization.

We reduced the number of variables to avoid overfitting and to decrease computational memory requirements. The number of admissions (i.e. samples) and variables were *n* = 27,334 and *p* = 34,621, respectively. As such, the number of variables p was much larger than the number of samples n. Such a situation where p> > n may lead to overfitting [18]. Additionally, a substantially high amount of GPU memory is required to train a deep-learning based predictive model with a large number of variables. To reduce the number of variables (p), we used the total Kullback-Leibler (KL) divergence method to evaluate the differences between the 30-day readmission group and the non-30-day readmission group [19]. Total KL divergence measures the difference between two distributions of a variable. If the two distributions are similar, the value of total KL divergence is small, and the redundant variable can be removed. Variables with a total KL divergence equal to or higher than the mean + 1/2 × standard deviation of all variables were retained for use in the prediction models, resulting in a final total of 3512 variables.

### Deep unified networks (DUNs)

**Fig. 1:**
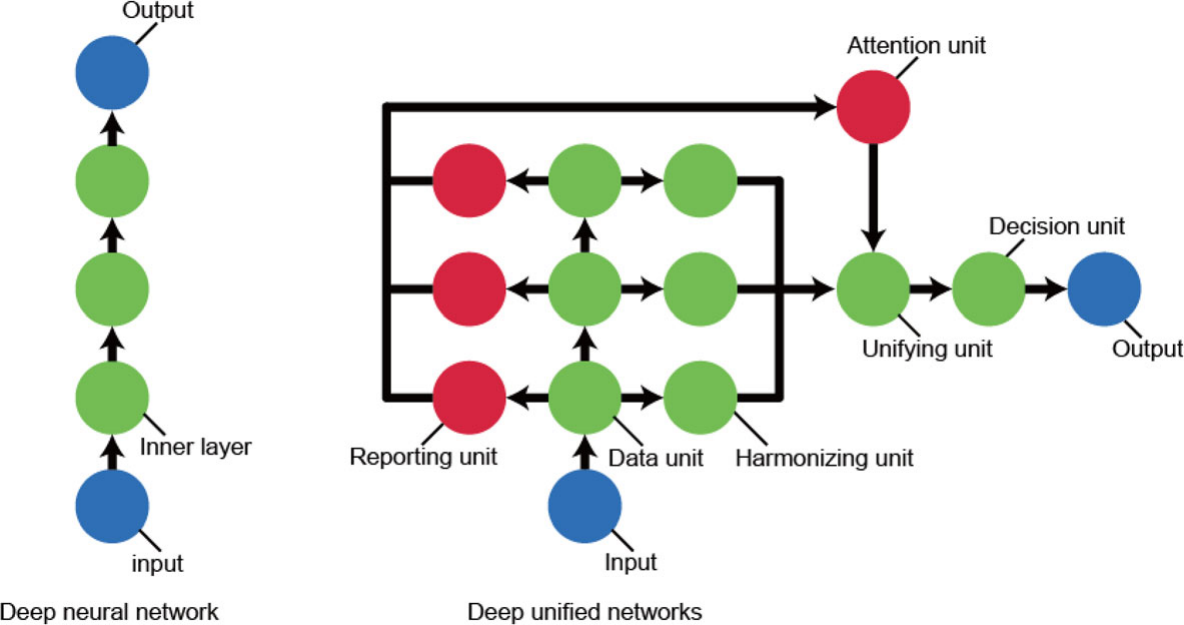
Network architectures of deep neural network and deep unified networks. Demonstrates the network architecture of deep unified networks (right side) compared to deep neural networks (left side)

We used artificial neural networks (NN), also known as deep learning, to build the prediction models. Ravì et al. introduced six different deep learning architectures: deep neural network (DNN), deep autoencoder, deep belief network, deep Boltzmann machine, recurrent neural network, and convolutional neural network (CNN) [20]. Generally, DNN is used for the task of early readmission prediction [21, 22]. In the case of the DNN architecture, it is difficult to train the lower inner layers [20]. Yan et al. showed that the HF readmission prediction performances of logistic regression and DNNs were almost equivalent (logistic regression: AUC 0.657 and DNN 0.662) [22]. So far, it has not been possible to use the DNN architecture successfully in the areas of computer vision and natural language processing, where CNNs have shown impressive results. Addressing this problem, *deep unified networks* (DUNs) are a newly developed architecture of deep learning characterized by the binding of each network layer’s neurons in a mesh-like form as shown in Fig. 1. The architecture of DUNs is different from the six conventional architectures of deep learning described above. Using vertical and horizontal connections of neurons, all inner layers of DUNs can learn the prediction task from the training data to avoid over-fitting. The DUNs architecture has horizontally shallow and vertically deep layers to prevent gradient vanishing and explosion. No matter how many layers deep the architecture is vertically, there are only two horizontal layers from the data unit nodes to the output node as shown in Fig. 1. Only the harmonizing and decision units have learning parameters. If a linear relationship can explain feature variables and 30-day readmissions, a deep learning architecture that has many layers is not suitable to avoid overfitting. On the other hand, if there is a nonlinear relationship between them, linear models such as logistic regression cannot predict the patient’s risk of readmission with high accuracy. The DUNs’ attention unit selects the appropriate inner layers (i.e., data units) depending on the complexity of the data available. For a more detailed explanation of how DUNs were applied in this present work, please see Additional file 1: Appendix A.

### Evaluation methodology

#### Prediction accuracy validation

The readmission prediction models were built using logistic regression, gradient boosting and maxout networks (existing deep learning), and DUNs (the proposed method described above). [18]. *Gradient boosting* is an ensemble learning composed of weak prediction models (decision trees in many cases) [18]. A software library of XGBoost for gradient boosting has a suitable balance between calculation speed and predictive performance [23]. *Maxout networks* are network architectures having neurons grouped into each inner layer. The architecture of maxout networks covers a standard DNN with dropout. When the number of maxout is one, the maxout networks are equivalent to DNN that was used by the previous studies of early readmission prediction tasks [21, 22]. Maxout networks empirically have higher predictive performance as the number of maxout increases [24].

We used area under curve (AUC) to evaluate the effectiveness of each prediction model. AUC was determined by setting thresholds according to the probability for prediction of readmission within 30 days calculated from the test data, then calculating true positive (TP) and false positive (FP) ratios sequentially while shifting the threshold level serially. Where the threshold level at which $\sqrt{FP^2\kern0.5em +\kern0.5em {\left(1- TP\right)}^2}$ was minimal, the presence/absence of readmission within 30 days was labeled, followed by calculation of accuracy, precision, recall, and fl-measure (fl).

Machine learning techniques have tuning parameters (such as a number of neurons in deep learning) that are also called the hyper-parameters [18]. Note that the hyper-parameters do not contain the learning parameters. Using all data samples to tune hyper-parameters may cause overfitting. Nonlinear machine learning, including gradient boosting and deep learning, are at especially high risk of overfitting. In this study, hyper-parameter tuning and prediction model evaluation were carried out by sampling 10% of data at random from the entire dataset, followed by 100 iterations of optimization of each technique’s hyper-parameters with the use of the tree-structured parzen estimator of the hyperopt library [15]. In each iteration of hyper-parameter optimization, the mean AUC was calculated by 10-fold cross validation (10-fold CV) as an indicator of prediction accuracy. The predictive accuracy of each technique also was evaluated with 10-fold CV. The optimized results of hyper-parameters in each method are described in the “Hyper-parameter optimization results” section below.

#### Cost saving evaluation

PHS offers heart failure telemonitoring (Connected Cardiac Care Program (CCCP)) to heart failure patients with recurrent hospitalizations [25]. We evaluated the economic benefits that could be generated by using the prediction models for selecting CCCP enrollees based on readmission risk. Net savings from readmission reduction were calculated for each classification threshold based on the receiver operating characteristic (ROC) curve of each round of 10-fold CV using the following equation.

$$ {\displaystyle \begin{array}{l} Net\  savings\ from\ readmission\ reduction\\ {}= Total\ saved\ readmission\ cost- Total\ CCCP\ cost\\ {}= Readmission\ cost\  per\  patient\times Number\ of\ true\ positives\times CCCP\ response\ rate\\ {}- CCCP\ cost\  per\  patient\times Number\ of\ predicted\ positives\end{array}} $$

where *readmission cost per patient* and *CCCP cost per patient are* $9655 and $1500 respectively [25]. *CCCP response rate* is the possibility of successfully preventing a readmission by applying CCCP to a patient who would be readmitted within 30 days without CCCP, which is set to 50% [26]. *Number of predicted positives* is the number of index admissions that were classified as positive (i.e., 30-days readmission occurs) by the prediction model.

We derived the maximum *net savings from readmission reduction* and the accuracy at the corresponding classification threshold for each round of the 10-fold CV. Then the mean and the standard deviation of the maximum net savings and the accuracy were derived.

## Results

### Patient population

**Fig. 2:**
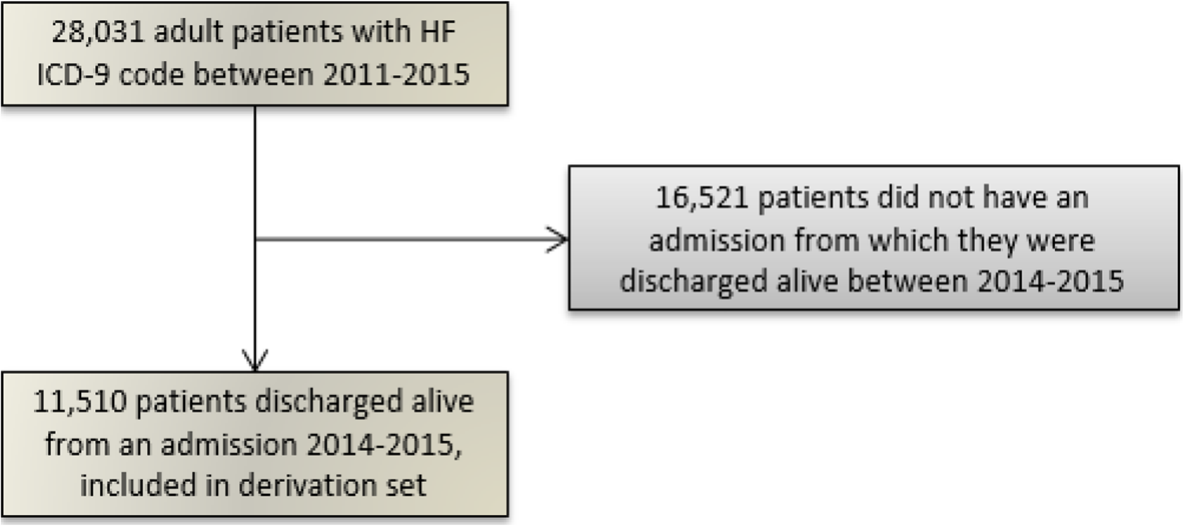
Patient selection flowchart. Summarizes the patient selection process

Figure 2 below summarizes the patient selection process. Of 28,031 patients identified as having a hospital encounter with HF as the principal diagnosis between 2011 and 2015, 11,510 (41%) were discharged alive from a hospital admission during the study period (between 2014 and 2015).

Table 2 below summarizes the patient demographics. The age distribution was skewed, with 75% of patients being 65 years of age or older. There were slightly more men (53%) than women (47%), and the cohort was primarily white (84.5%). A higher proportion of patients were married or partnered (45.6%), and roughly half the cohort (51.4%) had no higher than a high school education. The majority of patients (57.3%) were retired. Patients were also likely to be managing more than one health condition: 77.2% of patients presented to a hospital with one or more comorbidities during the study period, including hypertension (37.3%), other cardiovascular disease (32.5%), and chronic kidney disease (26.1%).

**Table 2:** Patient Cohort Demographics

Patient Characteristics	Whole Cohort	Patients with readmissions	Patients without readmissions	*p*-value
	*N* = 11,510	*n* = 3502	*n* = 8008	
Age Distribution				0.75
Median (1, 3 quartile)	75.7 (64.3, 84.7)	76.0 (64.4, 84.6)	75.5 (64.2, 84.7)	
Gender, n (%)				0.74
Male	6073 (52.8)	1839 (52.5)	4234 (52.9)	
Race, n (%)				0.16
White	9490 (84.5)	2872 (83.8)	6618 (84.9)	
Black or African American	889 (7.9)	297 (8.7)	592 (7.6)	
Hispanic or Latino (all races)	423 (3.8)	141 (4.1)	282 (3.6)	
Asian	221 (2.0)	61 (1.8)	160 (2.1)	
Other, or more than one race	202 (1.8)	58 (1.7)	144 (1.8)	
*Unknown (excluded in calculations)*	*n = 285*	*n = 73*	*n = 212*	
Marital status, n (%)				0.07
Married / Partnered	5125 (45.6)	1516 (44.3)	3609 (46.2)	
Widow	2360 (21.0)	718 (21)	1642 (21)	
Single	2274 (20.2)	715 (20.9)	1559 (19.9)	
Divorced / Separated	1069 (9.5)	323 (9.4)	746 (9.5)	
Other	412 (3.7)	148 (4.3)	264 (3.4)	
*Unknown (excluded in calculations)*	*n = 270*	*n = 82*	*n = 188*	
Highest educational attainment, n (%)				< 0.01*
Some High School or Less	1117 (12.7)	392 (14.2)	725 (12)	
High School Graduate/GED	3418 (38.7)	1127 (40.7)	2291 (37.8)	
Some College/Vocational/Technical Program	492 (5.6)	134 (4.8)	358 (5.9)	
Graduate of College or Postgraduate School	2911 (33.0)	864 (31.2)	2047 (33.8)	
Other	887 (10.1)	253 (9.1)	634 (10.5)	
*Unknown (excluded in calculations)*	*n = 2685*	*n = 732*	*n = 1953*	
Employment status, n (%)				< 0.01*
Retired	4338 (57.3)	1329 (57.7)	3009 (57.1)	
Employed ^a^	2067 (27.3)	556 (24.1)	1511 (28.7)	
Disability	656 (8.7)	259 (11.2)	397 (7.5)	
Unemployed	467 (6.2)	149 (6.5)	318 (6)	
Other	49 (0.6)	12 (0.5)	37 (0.7)	
*Unknown (excluded in calculations)*	*n = 3933*	*n = 1197*	*n = 2736*	
Number of comorbidities, n (%) ^b^				< 0.01*
0	2619 (22.8)	578 (16.5)	2041 (25.5)	
1	3032 (26.3)	840 (24)	2192 (27.4)	
2	2684 (23.3)	841 (24)	1843 (23)	
≥ 3	3175 (27.6)	1243 (35.5)	1932 (24.1)	

^a^Includes part-time and self-employment

^b^The following list of comorbidities was selected based on a literature review of comorbidities frequently found in patients with heart failure [27–33], in addition to clinical opinion of study staff physicians. Please see Additional file 3: Appendix B for a complete list of ICD-9 codes used to identify each condition. Each condition evaluated is listed here with the percentage of the study population who presented to a PHS facility with the condition as the principal diagnosis for either an inpatient or outpatient encounter at least once between 2014 and 2015. Hypertension (37.3%), cardiovascular disease (32.5%), chronic kidney disease / renal insufficiency (26.1%), non-secondary diabetes mellitus (22.8%), anemia (19.2%), chronic obstructive pulmonary disease (9.8%), osteoarthritis (9.1%), mental health conditions (7.2%), back pain (3.3%), osteoporosis (3.2%), obesity (2.3%)

In comparing patients who did (30.4%) versus did not (69.6%) have readmissions, we see the two groups did not differ significantly in age (*p* = 0.75), gender (*p* = 0.74), race (*p* = 0.16), or marital status (*p* = 0.07). However, they did differ significantly in level of education, employment status, and number of comorbidities (all *p* = < 0.01). Specifically, patients with readmissions were less likely to have had education above high school (*p* = < 0.01), less likely to be employed (*p* = < 0.01) and more likely to be on disability (*p* = < 0.01), and more likely to have ≥3 comorbid conditions.

### Inpatient hospital utilization by disease group

For the following analysis, we grouped the principal ICD-9 codes by disease category according to designations by the Health Cost and Utilization Project (HCUP) Clinical Classifications Software (CCS), which characterizes diagnoses into clinically meaningful categories making it easier to see diagnosis data patterns [34]. The top 10 diagnosis categories for the 27,334 inpatient admissions in descending order were as follows: HF (non-hypertensive; 22.3%), dysrhythmia (5.3%), septicemia (3.9%), device complications (3.7%), pneumonia (4.6%), hypertension (with complications; 3.3%), COPD (2.8%), acute renal failure (2.5%), urinary tract infection (2.2%), and acute myocardial infarction (2.1%). Of the device complications, 43% were related to cardiac devices.

### Readmission rates

To account for the likelihood of a readmission for HF regardless of index admission cause, we allowed for all-cause index admissions in our dataset. Of 27,334 inpatient admissions, 6374 (23.3%) were associated with 30-day readmissions. Of these 6374 admissions with a readmission, 1448 (22.7%) of the index admissions were flagged as HF-related, and 1461 (22.9%) of the readmissions were flagged as HF-related, with an overlap of 552 (8.7%) where both the index and its readmission were HF-related. Therefore, of the 1461 HF-specific readmissions, only 38% had index admissions which were HF-related.

### Common medications, labs, and procedures

The top 5 most commonly prescribed medications for this population during the study period, determined by percentage of patients with a prescription record for the drug anytime between 2014 and 2015, were Furosemide (60% of patients), Metoprolol (43%), Aspirin (43%), Omeprazole (39%), and Lisinopril (37%).

The top 5 most commonly ordered lab tests for this population during the study period, determined by a lab order date occurring during the target window described in Table 1, were electrolyte/renal/glucose panels (e.g. estimated glomerular filtration rate, glucose, potassium; 25% of labs), complete blood counts (e.g. hematocrit, white blood cell, red blood cell; 23%), blood differential absolute/percentage (e.g. nucleated red blood cell, lymph, eosinophil; 18%), general chemistries (e.g. calcium, magnesium, phosphorous; 8%), and routine coagulation (e.g. prothrombin time, international normalized ratio, partial thromboplastin time; 4%).

The top 5 most commonly performed principal procedures for this population during the study period, determined by a procedure date occurring between admission and discharge date, were procedures of the cardiovascular system (e.g. hemodialysis, cardiac catheter, coronary artherectormy; 36% of procedures), miscellaneous diagnostic and therapeutic procedures (e.g. non-invasive mechanical ventilation, packed cell transfusion, heart ultrasound; 29%), procedures of the digestive system (e.g. small bowel endoscopy, abdominal paracentesis, esophagogastroduodenoscopy; 13%), procedures of the musculoskeletal system (e.g. knee replacement, hip replacement, arthrocentesis; 9%), and procedures of the respiratory system (e.g. thoracentesis, endoscopic bronchial biopsy, insertion of intercostal catheter, 5%).

### Feature category variables

After reducing features via total KL divergence (method described above under Feature Variable Coding), the final algorithm included 3512 variables represented over 25 feature categories derived from demographic, admissions, diagnosis, procedure, medication, lab, and unstructured text data. Table 3 shows the number of feature categories and variables per category for each data type.

**Table 3:** Description of contributing variables and results of variable reduction post-processing, by data type

Data Type	Major Variables	Number of feature categories	Variable reduction from ➔ to
Demographics	Marital status, education, gender, language	2	39 ➔ 15
Admissions	Total cost of index admission, age at admission, cumulative number of 30-day readmissions, length of stay	2	217 ➔ 53
Diagnoses	ICD-9 codes, WKF	4	8101 ➔ 1297
Labs	WKF at admission, WKF at discharge	2	94 ➔ 58
Medications	RXCUI, Medication name, WKF	7	16,779 ➔ 1107
Procedures	ICD-9 codes	1	1833 ➔ 95
Notes	Words from: social history, hospital course, hospital reason, allergies	7	7558 ➔ 887
*Total*		*25*	*34,621* ➔ *3512*

### Prediction model evaluation

#### Hyper-parameter optimization results

The hyper-parameters for the logistic regression were the coefficient λ of the L1 or L2 norm of the weights, used to avoid multicollinearity. The logistic regression models with L1 and L2 norm regularization are equivalent to lasso and ridge regression models, respectively [18]. For hyper-parameter tuning, λ was varied in the range [− 15, 0] over the log-uniform distribution (= exp. (lower, upper)). The best results were obtained using the L2-norm for λ = 6.931 × 10^− 6^ with AUC 0.628. In the case of gradient boosting, Table 4 shows each hyper-parameter of the XGBoost library and its range of adjustment. For the parameters not shown in Table 4, gradient boosting used the standard values given in the library. The best AUC of gradient boost was 0.639.

**Table 4:** Hyper-parameters of Gradient Boosting (XGBoost), Maxout networks, and DUNs

		GRADIENT BOOSTING (XGBOOST)		
Parameter name		Distribution and search range	Best parameter	
learning_rate		Log-uniform [−5.0, −0.5]	0.007	
max_depth		Discrete uniform [3, 25]	5	
min_child_weight		Discrete uniform [1, 10]	1	
n_estimators		Discrete uniform [100, 1000]	398	
gamma		Log-uniform [−10, 0]	0.042	
alpha		Log-uniform [−10, 0]	0.0003	
lambda		Log-uniform [−10, 0]	0. 116	
subsample		Discrete uniform (units of 0.05) [0.5, 1.0]	0.70	
colsample_bytree		Discrete uniform (units of 0.05) [0.5, 1.0]	0.80	
		MAXOUT NETWORKS and DUNs		
Parameter name		Distribution and search range	Best parameter	
			*Maxout networks*	*DUNs*
Number of epochs		Discrete uniform [20, 100]	22	100
Number of inner layers		Discrete uniform [2, 5]	3	5
Number of inner neurons		Discrete uniform [100, 1000]	914	759
Number of maxout		Discrete uniform [2, 5]	5	–
Activation function		Random choice from: sigmoid, tanh, softplus, softsign	Sigmoid	Sigmoid
Dropout rate of:	- input layer	Uniform [0.001, 0.5]	0.446	0.397
	- inner layers	Uniform [0.001, 0.5]	0.394	0.433

Table 4 also shows the hyper-parameters of maxout networks and DUNs. Stochastic Gradient Descent (SGD) used a learning rate of 0.01 with momentum 0.9, and a batch size 100. Maxout networks selected three inner layers with 914 neurons. The inner layers and neurons of DUNs corresponded to those of the data unit, respectively. DUNs selected five layers with 759 neurons. Number of maxout is the number of inner layer groups. The best number of the groups was three. Activation function is the type of activation function for the inner layer of maxout networks, as well as the data units and harmonizing units of DUNs. Maxout networks and DUNs selected the sigmoid function. Dropout rate is a parameter of the dropout ratio for the input and the inner layers. Maxout networks and DUNs selected about 40% defect rate for the input and inner layers. The best AUC for maxout networks and DUNs were 0.624 and 0.636 respectively.

#### 10-fold cross validation results

**Fig. 3:**
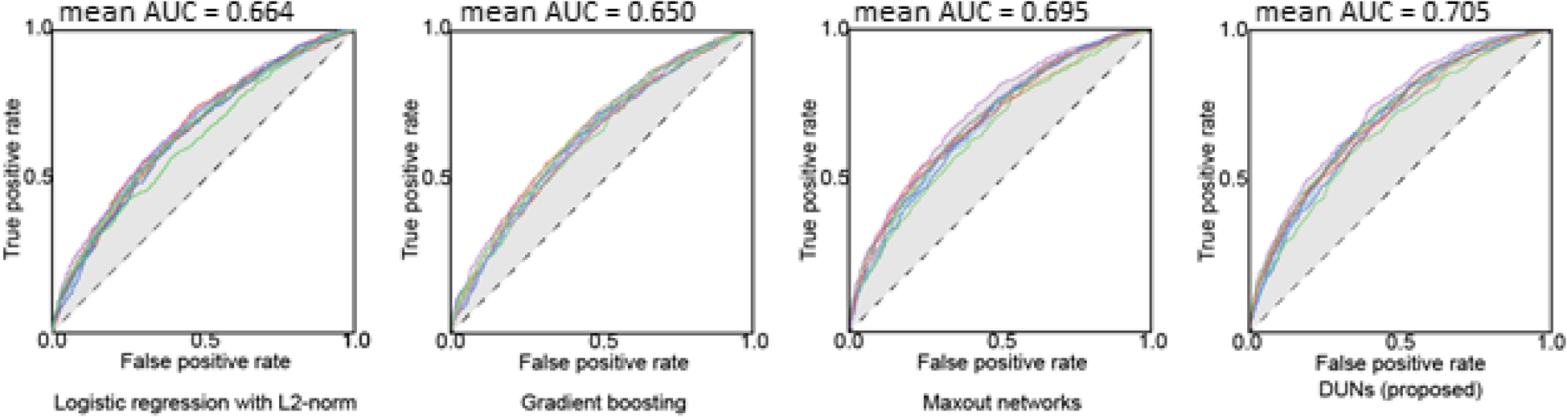
ROC curves of 10-fold CV. Demonstrates the ROC curve for each predictive modeling technique

Table 5 shows the mean ± standard deviation (std.) for 10-fold CV yielded from evaluation with each technique. The mean AUCs in Table 5 were calculated from the area under the receiver operating characteristic (AUROC) curves shown in Fig. 3, which displays the curve for each fold of the cross validation for each method. Only DUNs exceeded AUC 0.70 and marked the best value in each evaluation. The second-best result was obtained with maxout networks (AUC 0.695). There was no large turbulence in the ROC curve of DUNs, and the true positive rate rose slowly as the false positive rate rose with each technique. The AUC in the final 10-fold CV evaluation was improved from that in hyper-parameter tuning for all techniques. In the results of hyper-parameter optimization, gradient boosting marked the best performance (AUC 0.639), however, it was the worst performance (AUC 0.650) in the final 10-fold CV evaluation. On the other hand, with deep learning, the difference in AUC of 10-fold CV from that during hyper-parameter optimization was 0.071 higher for maxout networks and 0.069 higher for DUNs.

**Table 5:** 10-fold CV Results

	AUC mean ± sd	Accuracy mean ± sd	Precision mean ± sd	Recall mean ± sd	f1 mean ± sd
Logistic regression	0.664 ± 0.015	0.626 ± 0.020	0.336 ± 0.014	0.616 ± 0.029	0.435 ± 0.012
Gradient boosting	0.650 ± 0.011	0.612 ± 0.013	0.325 ± 0.008	0.615 ± 0.032	0.425 ± 0.010
Maxout networks	0.695 ± 0.016	0.645 ± 0.016	0.354 ± 0.016	0.631 ± 0.016	0.454 ± 0.016
DUNs (proposed)	0.705 ± 0.015	0.646 ± 0.018	0.360 ± 0.015	0.652 ± 0.036	0.464 ± 0.013

**Fig. 4:**
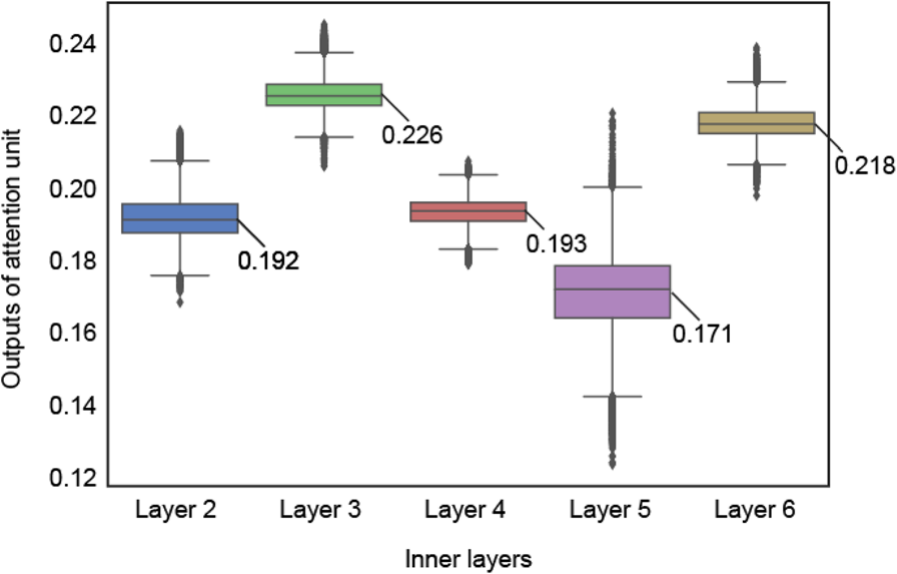
Layer importance of DUNs. Shows the boxplot distributions of the attention unit output (Y axis) against each layer number (X axis)

Figure 4 is a boxplot of the attention unit output for DUNs. As shown in Fig. 1, the attention unit generates weights to mix the output values obtained by the inner units for each input feature vector. The weights indicate the measure of difficulty of the 30-days readmission prediction task for each patient. DUNs calculate nonlinear features as a multiple feature combination by increasing the inner layer (i.e., data units). Thus, the upper layers’ features have a more complex nonlinearity than that of the lower layers. To improve prediction accuracy, DUNs’ attention unit selects the best features for each patient’s readmission case. For example, if a patient’s readmission prediction task does not have a nonlinear relationship between the input variables and the target variable, the attention unit generates strong weights for the lower inner layers. The mathematical mechanism of the attention unit is further described in Additional file 1: Appendix A.

Figure 4 plots the attention unit (0.0-1.0; Y axis) against the layer number (X axis). Layers yielding the strongest output were layer 3 (mean 0.226 ± std. 0.006) and layer 6 (mean 0.218 ± std. 0.005). The layer yielding the lowest output was layer 5 (mean 0.170 ± std. 0.011). The attention unit output from each layer ranged between 0.0 and 1.0, with the sum total of output from all layers being 1.0.

### Calculating importance of feature variables

Logistic regression and gradient boosting enable the calculation of the importance of feature variables, as determined by the frequency with which the variable was used for decision making during XGBoost. Because the AUC of logistic regression was higher than that of gradient boosting, we selected logistic regression to obtain the importance of feature variables. Logistic regression calculated the absolute values (magnitude) of the learning parameters (weight coefficients) as the feature importance. Table 6 shows the top 15 most important features in the logistic regression. More information about all feature variables (names, data types, importance) will be made available on our website as of July 2018 (HitachiPartnersAIproject.com).

**Table 6:** Feature importance ranking of logistic regression

Rank	Feature description
1	Cumulative number of *30-day readmissions*
2	Presence of *acute respiratory failure* ICD-9 code
3	Number of *abnormal sodium level* laboratory tests
4	Presence of *pneumonia (organism unspecified)* ICD-9 code
5	Number of *abnormal chloride level* laboratory tests
6	Presence of *acute kidney failure (unspecified)* ICD-9 code
7	Presence of *diabetes (with other specified manifestations)* ICD-9 code
8	Presence of *acute kidney failure (any)* ICD-9 code
9	Presence of *respiratory failure*, WKF
10	Presence of *disorders of fluid electrolyte and acid-base balance (any)* ICD-9 code
11	Presence of *disorders of fluid/electrolyte/acid-base*, WKF
12	Number of *abnormal albumin level* laboratory tests
13	Presence of *asphyxia and hypoxemia* ICD-9 code
14	Presence of *anemia of chronic illness* ICD-9 code
15	Presence of *hypokalemia* ICD-9 code

#### Cost evaluation results

**Fig. 5:**
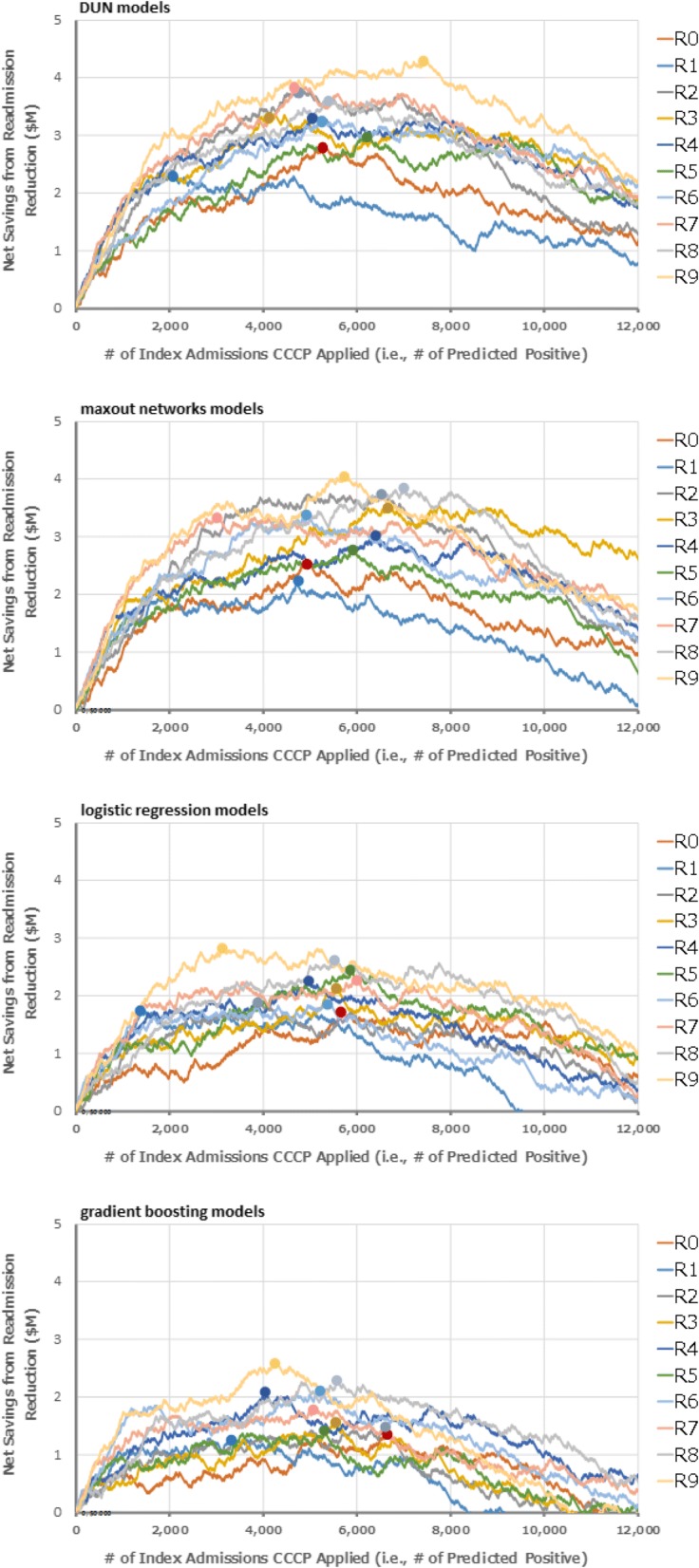
Projected net savings from readmission reduction by using prediction models to select CCCP enrollees. Shows the net savings from readmission reduction, calculated by changing the number of CCCP enrollees along with the classification threshold, using the ROC curves of 10-fold CV of 1) DUNs, 2) maxout networks, 3) logistic regression, and 4) gradient boosting as shown in Fig. 3

Figure 5 shows the net savings from readmission reduction, calculated by changing the number of CCCP enrollees along with the classification threshold, assuming the CCCP enrollees were selected for 2 years from 2014 to 2015, using the ROC curves of 10-fold CV of DUNs, maxout networks, logistic regression, and gradient boost models as shown in Fig. 3. The series of R0 to R9 represent the round number of the 10-fold CV. The 10 circles in the chart correspond to the point where the net savings reached the maximum in each round.

Table 7 shows the mean ± standard deviation of the maximum net savings and accuracy at the classification threshold corresponding to the maximum net savings which were derived from the 10-fold CV for each technique. DUNs demonstrated the maximum net savings at 3.403 ± 0.536 million. The mean ± standard deviation of the accuracy at the classification threshold that corresponds with the maximum cost savings was 76.4 ± 1.4%.

**Table 7:** Maximum net savings and corresponding accuracies

	Max net savings ($M)	Accuracy
DUNs (proposed)	3.403 ± 0.536	0.764 ± 0.014
Maxout networks	3.241 ± 0.561	0.754 ± 0.014
Logistic regression	2.173 ± 0.357	0.750 ± 0.018
Gradient boosting	1.787 ± 0.428	0.739 ± 0.016

## Discussion

This was a retrospective study which applied deep learning to structured and unstructured patient data from the EMR of a large healthcare delivery network to create a risk prediction model to predict 30-day readmissions in patients with HF. With an AUC of 0.705, the developed model performs moderately well, with results within the upper range compared with previously published models.

The DUNs AUC marked the best result of 10-fold CV compared to logistic regression, gradient boosting, and maxout networks. The mean AUCs in the final 10-fold CV for deep learning-based techniques were about 0.07 points higher compared to hyper-parameter optimization results. Hyper-parameter overfitting occurred in gradient boosting, as demonstrated by its having the highest AUC in the hyper-parameter optimization results but the lowest AUC in the final 10-fold CV results. This suggests that prediction models that have many hyper-parameters need a particular validation scheme to measure actual prediction accuracy.

For the prediction task of early readmission after hospitalization for HF, Futoma, et al. and Yang, et al. independently compared logistic regression with DNN using three inner layers [21, 22]. For the 10-fold CV results evaluated by Futoma, et al., the AUC of the DNN (0.676) was 0.022 points higher than that of the logistic regression (0.654) [21]. For the 10-times random split validation results evaluated by Yang, et al., the AUC of the DNN (0.662) was 0.005 points higher than that of the logistic regression (0.657) [22]. The 10-fold CV results in Table 5 show that the AUC of maxout networks and DUNs were, respectively, 0.031 and 0.041 points higher than that of logistic regression. This suggests that if deep learning-based techniques are applied to cases where a large training data set is available, the automated generation of feature variables – a capability unique to deep learning – works sufficiently, yielding higher accuracy when compared to the other techniques.

Because of the DUNs’ attention unit’s ability to maximize prediction accuracy by selecting an inner layer depending on the level of nonlinearity between the input variables and the target variable for each readmission case, the architecture of DUNs was more suitable compared to conventional DNNs and other traditional classifiers for this readmission prediction task, due to the dataset containing both linear and nonlinear relationships. The AUC of 0.665 achieved with logistic regression solved simpler cases i.e. cases where a linear relationship could explain feature variables and 30-day readmissions. The higher AUC of 0.695 achieved with maxout networks suggests the more complex cases had a nonlinear relationship. Maxout networks selected three inner layers (the optimization range is two from five), suggesting that high-dimensional feature variables with four or more layers are not necessary for all readmission cases. As shown in Fig. 4, the DUN attention unit weights were higher for layers three and six, indicating that the 30-day readmission events consisted of a mixture of cases which could be explained by the feature variables of the dimension similar to maxout networks, and cases which could be explained by the high-dimensional six-layer feature variables.

The calculated importance of feature variables (Table 6) highlighted a number of characteristics representative of symptoms related to HF and its frequent comorbidities. Four features pertained to pulmonary disease and distress (#2 and 9: respiratory failure ICD-9 code and WKF, #4: pneumonia ICD-9 code, and #14: asphyxia and hypoxemia ICD-9 code), all potentially symptomatic of COPD, which was present more frequently as a comorbidity in the patient segment with readmissions compared to those without (13% vs. 8.5%, *p* < 0.001). Eight pertained to renal disease and monitoring (#6 and 8: acute kidney failure ICD-9 codes, #3, 5, and 12: abnormal sodium, chloride, and albumin labs, #10 and 11: disorders of fluid/electrolyte/acid-base ICD-9 code and WKF, and #15: hypokalemia), the labs in particular all being part of a basic renal panel. Chronic kidney disease was also more represented in the patient segment with readmissions compared to those without (36.5% vs. 21.6%, *p* < 0.001). Similarly, features #7 and 14 – diabetes and anemia – were more represented in patients with readmissions compared to those without (respectively: 26.1 vs. 21.4%, *p* < 0.001; and 24.8 vs. 16.7%, p < 0.001).

Following external validation to assure efficacy, the developed model has promising clinical uses. Healthcare organizations and providers might consider using such a model to monitor their patients with HF, to assess their likelihood of readmission following discharge from an inpatient admission. Using predictive analytics to select high readmission risk patients to receive telemonitoring intervention may help to save on intervention costs to the hospital. Upon identification of readmission risk, the patient’s care team may consider an enhanced review of the patient’s current comorbidities and care plan at discharge, to determine if there are any care gaps which can be filled to potentially avoid readmission. Additionally, knowledge of one’s risk for readmission may also provide patients with important decision-making information about their own health, such as adopting health changes (e.g. diet, medication adherence) or increased communication with their care team.

### Strengths, limitations, and challenges

The model was developed on a HF patient population and included HF-specific WKFs as features, however, the index event itself need not be HF-specific. By including all-cause index as well as all-cause readmissions, a strength of this study may be increased generalizability of the model to any encounter the HF patient experiences. The readmission rates in this study suggest that in the event of a readmission, patients are as likely to be readmitted for HF regardless of index admission as they are to have an index HF admission. This finding corresponds to insights from previous studies. In an analysis of re-hospitalization patterns using Medicare claims data, Jencks et al. found that HF was the most frequent cause of readmission regardless of index admission diagnosis [35]. Similarly, Dharmarajan et al. found a high occurrence of HF readmissions regardless of index diagnosis in their analysis of Medicare claims data, and further emphasize that any admission places a patient in a position of heightened vulnerability to a variety of conditions throughout the post-discharge period [36]. In this study, the finding that *cumulative number of 30-day readmissions* is ranked first among logistic regression feature importance (Table 6) further suggests that any admission is itself a contributor to readmission risk. Thus, for patients with heart failure, focusing only on HF index admissions may result in missed opportunities to intervene to prevent any readmission, including that for HF itself. The remaining top-ranked 14 features, as discussed above, likewise highlight the importance of monitoring HF patients for their common comorbidities which also put them at risk for readmission.

Another strength of this study is the inclusion of both structured and unstructured clinical data. To our knowledge, this is the first model to apply deep learning to a readmission prediction algorithm for heart failure patients featuring both structured and unstructured data. By including both structured and unstructured data as features, the algorithm utilizes more robust data available on a given patient at the time of discharge, including important patient information not generally available in a structured format, such as patient’s current living situation (available as social history), and details about the patient’s course through the hospital during admission (available through hospital course and history of present illness).

The vertically deep and horizontally shallow architecture of DUNs prevented overfitting of both the hyper- and learning parameters, and constructed a high accuracy prediction model (AUC 0.705). However, DUNs do not mathematically calculate the importance of feature variables. Logistic regression provided the feature importance as shown in Table 6, although it could not achieve an AUC higher than 0.700. Thus, a limitation of deep learning, including DUNs, is the lack of ‘explainability’ of the prediction models [37, 38]. The DUNs’ prediction model might contain unknown and nonstandard knowledge which could possibly improve the treatment of HF patients. Future work will focus on developing explainable deep learning to provide tailored feedback to physicians.

There are limitations to the study. With regards to algorithm development, KL-divergence filtering - a supervised feature selection process - was the first step in our experiments, meaning the feature selection procedure had access to the entire dataset to pre-select a subset of features that were predictive of the outcomes, which could potentially lead to overfitting (i.e., an exaggerated performance). Some feature selection methods use supervised classifiers such as logistic regression and random forests. The classifier-based methods have much higher feature selection ability than the total KL-divergence filtering. There might be a trade-off between the feature selection ability and the risk of overfitting (i.e. accuracy decreasing in a test dataset). We used the total KL-divergence filtering as the conservative approach. Additionally, we used an external dataset to measure the overfitting effect of variable preprocessing and prediction model construction (described in Additional file 2: Appendix C). In this experiment, using the total KL-divergence method for feature selection did not lead to overfitting. The AUC of the 10-fold CV described above and of this external evaluation was 0.705 (as shown in Table 5) and 0.720, respectively. This suggests the DUNs demonstrate consistent prediction ability in this retrospective study. We will plan a prospective study to evaluate the use of the DUNs’ prediction model in our future research.

Missing data is a well-known limitation of utilizing EMR data for research [8]. Specifically within the PHS network, until 2014, various facilities used separate EMR systems, which collect data in different ways. Since 2014, facilities have updated their EMR to a single system, and each facility adopted this new EMR on their own schedule. This means that data was collected for patients in multiple ways across multiple systems over several years. The treatment of missing values is described above under Feature Variable Coding. An important next step for this work is to validate the algorithm on current data now that all PHS network facilities are on the same EMR system. The increased consistency of data collected within a single EMR system may have an effect on the performance of the model.

An additional limitation is that the data contains only episodes of care which occurred within the PHS network, meaning any admissions to out-of-network facilities are not captured in the data. Relatedly, results may not be generalizable to other healthcare systems, due to specifics of the patient population demographics (see Table 2) and the nature of being a large integrated healthcare delivery network.

Future work will consider the use of scaled exponential linear unit (SELU), a recently developed activation technique [39], to examine its effect on the performance of neural networks. Additionally, to improve the prediction accuracy of DUNs, future work may consider other techniques of deep learning such as activation functions (e.g. SELUs with alpha-dropout [39]), normalization techniques (ext. batch, weight, and layer normalization [40]), and faster SGDs (e.g. YellowFin [41], entropy-SGD [42]).

## Conclusions

The value of this model is its ability to identify heart failure patients with an impending hospitalization regardless of cause of index admission. This enables care teams to target interventions at their most high-risk HF patients and improve overall clinical outcomes by treating the whole picture of the patient, not just a single diagnosis, which is important in a population with high rates of comorbidity and readmission. In the next stage of this project, we aim to validate this model on a new set of patient data. Like the present study, this new data set will utilize data generated by PHS patients, but will exclude patient data generated by patients whose data was included as part of algorithm development or the experiment described in the limitations above and in Additional file 2: Appendix C. Data will also be more recent, being generated from mid-2017 to present, to assure all data was collected within a single EMR system, as discussed in the limitations section above.

Additionally, we will conduct a multi-stage feasibility and usability study which will examine in detail what inputs, sources, computational requirements, and cost are necessary to functionally implement a predictive tool such as the one described herein into care provider workflow. The evaluation will include interviews with key stakeholders to determine perceived clinical value and relevance, desired output and user interface, and how the tool will be integrated into clinical workflow. Finally, we will conduct a comprehensive impact assessment to better understand the potential cost savings and value addition of the tool to an integrated healthcare network.

Successful implementation of a readmission risk prediction model could give care teams valuable insights to their patient pool, identifying high-risk patients and permitting the opportunity to target early clinical interventions to these patients with the aim of reducing the likelihood of readmission.

## Additional files

Additional file 1: Appendix A. Deep Unified Networks, supplemental. (DOCX 28 kb)

Additional file 2: Appendix C. External Dataset Evaluation to Measure Overfitting. (DOCX 23 kb)

Additional file 3: Appendix B. ICD-9 codes for identifying comorbid conditions. (DOCX 19 kb)

## Abbreviations

ACCF: American College of Cardiology Foundation
AHA: American Heart Association
ALT: Alanine Aminotransferase
ATC: Anatomical Therapeutic Chemical
AUC: Area Under the [Receiver Operating Characteristic] Curve
AUROC: Area Under the Receiver Operating Characteristic
BNP: Brain Natriuretic Peptide
BOW: Bag of Words
CCCP: Connected Cardiac Care Program
CCS: Clinical Classifications Software
CMS: Center for Medicare & Medicaid Services
CNN: Convolutional Neural Network
COPD: Chronic Obstructive Pulmonary Disease
CV: Cross Validation
DNN: Deep Neural Network
DUN: Deep Unified Network
EDW: Enterprise Data Warehouse
EMR: Electronic Medical Record
HCUP: Health Cost and Utilization Project
HF: Heart Failure
HIPAA: Health Insurance Portability and Accountability Act of 1996
HRRP: Hospital Readmissions Reduction Program
ICD: International Classification of Diseases
IRB: Institutional Review Board
KL: Kullback-Leibler
LOINC: Logical Observation Identifiers Names and Codes
NLP: Natural language processing
NN: Neural Network
NT-proBNP: N-terminal pro b-type natriuretic peptide
PHS: Partners HealthCare System
POS: Parts of Speech
ROC: Receiver Operating Characteristic Curve
RPDR: Research Patient Data Repository
SELU: Scaled Exponential Linear Unit
SGD: Stochastic Gradient Descent
SQL: Sequential Query Language
SSL: Secure Sockets Layer
SSMS: Microsoft SQL Server management Studio
WKF: Well-Known Factors
