# Supplementary material for: A machine learning model to predict the risk of 30-day readmissions in patients with heart failure: a retrospective analysis of electronic medical records data
Source: BMC Med Inform Decis Mak. 2018 Jun 22;18:44. doi: 10.1186/s12911-018-0620-z (PMC6013959; doi:10.1186/s12911-018-0620-z)
Supplement: Supplementary file 1 — Appendix A. Deep Unified Networks, supplemental. (DOCX 28 kb) [file 12911_2018_620_MOESM1_ESM.docx]

**Additional file 1. Deep Unified Networks, supplemental**

This appendix offers a detailed explanation of how deep unified networks (DUNs) were applied in the work described in the manuscript titled “A Machine Learning Model to Predict the Risk of 30-day Readmissions in Patients with Heart Failure: a Retrospective Analysis of Electronic Medical Records Data”. Figure 1 from the manuscript, demonstrating a comparison of the network architectures of DUNs and deep neural networks, is presented below for reference.

[INSERT FIGURE 1- Title: Network architectures of deep neural network and deep unified networks]

Artificial NNs were used to build the prediction models. Let $\boldsymbol{x}_{\boldsymbol{n}}\in\mathcal{R}^{d} (n=1, \cdots, N)$ represent a feature vector containing the $d$-dimensional patient information, with N denoting the sample size and $\mathcal{R}$ indicating the real number set. Let $t_{n}\in\left\{ 0, 1 \right\}$be the target label expressing the presence/absence of readmission within 30 days. Let $y_{n}\mathcal{\in R}^{d} (0\leq y_{n}\leq1 )$ be the predicted value. The total-binding type of the NN in Figure 1 (left side) of $L (l=1, \cdots, L)$ layers can be expressed as follows:

$y_{n}\boldsymbol{=}f^{L}g^{L}\boldsymbol{\cdots}f^{l}g^{l}\boldsymbol{\cdots}f^{2}g^{2}f^{1}g^{1}\left( \boldsymbol{x}_{n} \right),$ (1)

where $f^{l}$ is an activation function, and the function $g^{l}$ is defined as $g^{l}\left( \boldsymbol{x} \right)=\boldsymbol{Wx (W}\in\mathcal{R}^{d^{l+1}\times d^{l}})$ where $\boldsymbol{W}$ is a learning parameter. For simplicity, biased parameters have been removed from Eqn. (1). The pair of functions $f^{l}$and $g^{l}$ are called “the inner neurons of the $l$-th layer.” Prediction of readmission within 30 days was treated as a binary classification problem, and the last layer’s activation function, $f^{L}$, was deemed as the sigmoid activation function. The function given in Eqn. (1) is known as likely to result in overfitting if the number of layers $L$ is increased [18]. A possible factor responsible for this phenomenon is the progression of learning confined to the upper layer parameter $\boldsymbol{W}^{L}$and lack of advanced learning in the lower layers.

To address this problem, deep unified networks (DUNs) was developed as a new architecture of deep learning characterized by the binding of each layer’s neurons in a mesh-like form. Figure 1 (right side) shows the network architecture of DUNs. DUNs have data units as neurons corresponding to the neurons of each layer of the conventional NN. The data units are defined by the following equation:

$$\boldsymbol{h}_{D}^{l+1}\boldsymbol{=}f_{D}^{l}g_{D}^{l}\left( \boldsymbol{h}_{D}^{l} \right)\boldsymbol{,} (2)$$

where $\boldsymbol{h}_{D}^{l}\in\mathcal{R}^{d^{l}}$represents the input/output of the data units. $f_{D}^{l}$is an activation function. $g_{D}^{l}$ is a function representing learning parameters. In our study, highway networks were employed [43]. Note that when $l=1,$ we assumed$\boldsymbol{h}_{D}^{1}=\boldsymbol{x}_{n}$ and $g_{D}^{1}\left( \boldsymbol{x} \right)=\boldsymbol{Wx (W}\in\mathcal{R}^{d^{l=2} \times d})$.

We considered the data unit vector $\boldsymbol{h}_{D}^{l}$ are merged by using weighted averaging in the use of the unifying unit described below (Eqn. (6)). Since the vector size $d^{l}$ may be different for each *l*-layer, the output vectors $\boldsymbol{h}_{D}^{l}$ of data unit cannot be averaged in the unifying unit. To resolve this issue, for each data unit, the harmonizing unit converts the vector $\boldsymbol{h}_{D}^{l}$ to the vector $\boldsymbol{h}_{H}^{l}$ in the same feature space $\mathcal{R}^{h}$ for the weighted averaging in the unifying unit. The Harmonizing units are defined by the following equation:

$$\boldsymbol{h}_{H}^{l}\boldsymbol{=}f_{H}\left( \boldsymbol{W}_{H}^{l}\boldsymbol{h}_{D}^{l} \right), (3)$$

where $\boldsymbol{W}_{H}^{l}\in\mathcal{R}^{h\times d^{l}} \left( h\leq d^{l} \right)$ is a learning parameter and $f_{H}$is an activation function. The harmonizing units bind to the data units when $2\leq l$.

To extract the abstract information from the data units vector $\boldsymbol{h}_{D}^{l}$, reporting units compress the $d^{l}$-dimensional vector $\boldsymbol{h}_{D}^{l}$ to an $r$-dimensional vector $\boldsymbol{h}_{R}^{l}$. The Reporting units are defined by the following equation:

$$\boldsymbol{h}_{R}^{l}\boldsymbol{=}\sigma\left( \boldsymbol{W}_{R}^{l}\boldsymbol{h}_{D}^{l} \right), (4)$$

where$\boldsymbol{W}_{R}^{\boldsymbol{l}}\in\mathcal{R}^{r\times d^{l}} \left( r\leq d^{l} \right)$ is a learning parameter, and $\sigma$ is a sigmoid function. The reporting units bind to the data units when $2\leq l$. In our study, $r=1$ was used.

An attention unit calculates the *L*$-$1-dimensional weight vector $\boldsymbol{\alpha}$ as the weights of the data unit vector $\boldsymbol{h}_{D}^{l}$ for the unifying unit. The Attention unit is defined by the following equation:

$$\boldsymbol{\alpha=}\mathrm{softmax}\left( \boldsymbol{W}_{A}\boldsymbol{[h}_{R}^{2}\boldsymbol{, \cdots,}\boldsymbol{h}_{R}^{L}\boldsymbol{]} \right), (5)$$

where $\boldsymbol{W}_{A}^{l}\in\mathcal{R}^{L-1 \times m} (m=r\left( L-1 \right))$ is a learning parameter, and the softmax function serves as an activation function. The vector $\boldsymbol{[h}_{R}^{2}\boldsymbol{, \cdots,}\boldsymbol{h}_{R}^{L}\boldsymbol{]}$ is a *m*-dimensional vector yielded from stacking of individual vectors.

The unifying unit calculates the weighted average of the harmonized vector $\boldsymbol{h}_{H}^{l}$ as the following equation:

$$\boldsymbol{h}_{M}\boldsymbol{=}\sum_{k=1}^{L-1} \boldsymbol{\alpha[}k\boldsymbol{]}\boldsymbol{h}_{H}^{k+1}, (6)$$

where $\boldsymbol{\alpha[}k\boldsymbol{]}$ is an element of the *k*-dimension of weight vector $\boldsymbol{\alpha}$. Finally, the decision unit is a neuron which calculates the probability of readmission:

$$y_{n}\boldsymbol{=}\sigma\left( \boldsymbol{W}_{O}\boldsymbol{h}_{M} \right)\boldsymbol{, (}7\boldsymbol{)}$$

where $\boldsymbol{W}_{O}\in\mathcal{R}^{1\times h}$ is a learning parameter. The input vector $\boldsymbol{x}_{n}$ passes through one of the harmonizing units and the decision unit. Thus, the number of inner layers of DUNs is interpreted as the number of data units$(L-1$) + 2. In conclusion, there are 6 types of neuron constituting the DUNs. The learning parameters for all neurons were optimized to minimize the negative log likelihood loss of output $y_{n}$ for the target label $t_{n}$ (Eqn. (7) [18]).

**Reference**

1. Srivastava, R. K., Greff, K., & Schmidhuber, J. Highway networks. arXiv preprint arXiv:1505.00387. 2015.
