# Supplementary material for: A machine learning model to predict the risk of 30-day readmissions in patients with heart failure: a retrospective analysis of electronic medical records data
Source: BMC Med Inform Decis Mak. 2018 Jun 22;18:44. doi: 10.1186/s12911-018-0620-z (PMC6013959; doi:10.1186/s12911-018-0620-z)
Supplement: Supplementary file 2 — Appendix C. External Dataset Evaluation to Measure Overfitting. (DOCX 23 kb) [file 12911_2018_620_MOESM2_ESM.docx]

**Additional file 2. External Dataset Evaluation to Measure Overfitting**

We used an external dataset to evaluate the presence of overfitting that may have resulted from variable preprocessing and prediction model construction. The external dataset consisted of data from a random selection of 90 patients who were admitted within PHS hospitals from Jan 2017 - May 2017. Patients were selected using the same inclusion and exclusion criteria as that of the primary dataset, with the added criteria that they not have had data which was included in the primary dataset. Out of 90 index admission events, 30 were followed by 30-day readmissions. We did not balance the ratio of presence/absence of 30-day readmissions. The prediction models of 10-fold CV, evaluated in the Prediction Model Evaluation section described in the manuscript, composed a single prediction model for the external evaluation (from here on referred to as the “external prediction model”). The external prediction model outputs the averaged probability of 30-day readmission calculated by the prediction models of 10-fold CV.

The DUNs marked the highest AUC compared to logistic regression, gradient boosting, and maxout networks as shown in Table S8. The mean AUCs of the 10-fold CV and the external evaluation for all prediction models were 0.6785 and 0.682, respectively. The DUNs' AUC was 0.15 points higher compared to the 10-fold CV results of the primary dataset. The second-best model was the logistic regression, and the maxout networks moved down from second to third. The gradient boosting lost 0.015 points from the 10-fold CV results.

The total KL-divergence filtering has a weaker feature selection ability compared to that of other methods that use classifiers such as logistic regression and random forests. As compared in Table 5 and S8, the differences of the 10-fold CV and the external evaluation did not demonstrate overfitting as a result of feature selection. The weakness of the total KL-divergence filtering could not induce overfitting. Notably, the DUNs marked the highest AUCs in both evaluations. This suggests the consistency of DUNs’ prediction ability in this retrospective study. However, the number of patients in the external dataset was small (n = 90). We will plan a prospective study to evaluate the use of the DUNs' prediction model in our future research.

Table S8. External dataset evaluation results (n = 90)

|  | **AUC** | **Accuracy** | **Precision** | **Recall** | **f1** |
| --- | --- | --- | --- | --- | --- |
| Logistic regression | 0.699 | 0.656 | 0.333 | 0.550 | 0.415 |
| Gradient boosting | 0.635 | 0.611 | 0.325 | 0.700 | 0.444 |
| Maxout networks | 0.673 | 0.644 | 0.324 | 0.550 | 0.407 |
| DUNs (proposed) | 0.720 | 0.667 | 0.368 | 0.700 | 0.483 |
