# Supplementary material for: A machine learning model to predict the risk of 30-day readmissions in patients with heart failure: a retrospective analysis of electronic medical records data
Source: BMC Med Inform Decis Mak. 2018 Jun 22;18:44. doi: 10.1186/s12911-018-0620-z (PMC6013959; doi:10.1186/s12911-018-0620-z)
Supplement: Supplementary file 3 — Appendix B. ICD-9 codes for identifying comorbid conditions. (DOCX 19 kb) [file 12911_2018_620_MOESM3_ESM.docx]

**Additional file 3. ICD-9 codes for identifying comorbid conditions**

The supplemental table below outlines the ICD-9 codes used to identify comorbid condition groups used to characterize the patient population described in the manuscript titled “A Machine Learning Model to Predict the Risk of 30-day Readmissions in Patients with Heart Failure: a Retrospective Analysis of Electronic Medical Records Data”.

| **Condition** | **ICD-9 Code** | **Code Description** |
| --- | --- | --- |
|  |  |  |
| Hypertension |  |  |
|  | 401.xx–405.xx | Hypertensive Disease |
|  |  |  |
| Chronic Kidney Disease /  Renal Insufficiency |  |  |
|  | 403.xx | Hypertensive chronic kidney disease |
|  | 404.xx | Hypertensive heart and chronic kidney disease |
|  | 584.x | Acute kidney failure |
|  | 585.x | Chronic kidney disease |
|  | 586 | Renal failure, unspecified |
|  | 587 | Renal sclerosis, unspecified |
|  | 588.x | Disorders resulting from impaired renal function |
|  |  |  |
| Diabetes Mellitus |  |  |
|  | 250.xx | Diabetes mellitus |
|  |  |  |
| Obesity |  |  |
|  | 278.x | Overweight, obesity and other hyperalimentation |
|  |  |  |
| Cardiovascular Disease |  |  |
|  | 410.x x–414.xx | Ischemic Heart Disease |
|  | 415 | Acute cor pulmonale |
|  | 394.x–396.x | Valvular diseases (mitral, aortic, mitral aortic) |
|  | 424.x | Other diseases of endocardium |
|  | 746.x | Other congenital anomalies of heart |
|  |  |  |
| Osteoporosis |  |  |
|  | 733.0x | Osteoporosis |
|  |  |  |
| Back Pain |  |  |
|  | 724.1 | Pain in thoracic spine |
|  | 724.5 | Backache |
|  |  |  |
| Arthritis |  |  |
|  | 715.xx | Osteoarthrosis and allied disorders |
|  | V13.4 | Personal history of arthritis |
|  |  |  |
| Mental Health Conditions |  |  |
|  | 296.xx | Episodic mood disorders |
|  | 300.xx | Anxiety, dissociative / somatoform disorders |
|  | 309.xx | Adjustment reaction |
|  | 311 | Depressive disorder, not elsewhere classified |
